# Surface Correlation-Based Fingerprinting Method Using LTE Signal for Localization in Urban Canyon

**DOI:** 10.3390/s19153325

**Published:** 2019-07-29

**Authors:** Jung Ho Lee, Beomju Shin, Donghyun Shin, Jinwoo Park, Yong Sang Ryu, Deok Ha Woo, Taikjin Lee

**Affiliations:** 1 Sensor System Research Center, Korea Institute of Science and Technology, 5, Hwarang-ro 14-gil, Seongbuk-gu, Seoul 02972, Korea; 2 Department of Electrical and Computer Engineering, Korea University, Anam-dong, Seungbuk-gu, Seoul 136-713, Korea

**Keywords:** localization, fingerprinting, LTE, correlation, pedestrian, PDR

## Abstract

The Global Satellite Navigation System (GNSS) used in various location-based services is accurate and stable in outdoor environments. However, it cannot be utilized in an indoor environment because of low signal availability and degradation of accuracy due to the multipath distortion of satellite signals in urban areas. On the contrary, LTE signals are available almost everywhere in urban areas and are quite stable without much variation throughout the year. This is because of the fixed location of base stations and the well-maintained policy of mobile communication service providers. Its varied stability and reliability make LTE signals a more viable method for localization. However, there are some complexities in utilizing LTE signals including signal interference distortion phenomena during propagation multipath fading, and various types of noise. In this paper, we propose a surface correlation-based fingerprinting method to utilize LTE signals for localization in urban areas. The surface correlation converts timely measured signal strength into spatial pattern using the walking distance from a Pedestrian Dead-Reckoning (PDR). The surface correlation is carried out by comparing the spatial signal strength pattern of a pedestrian`s movement trajectory with a fingerprinting database to estimate the location. A reference trajectory of the moving pedestrian is chosen to have a greater correlation among the multiple trajectory candidates generated from a link-based fingerprinting database. By comparing spatial signal strength patterns, the proposed method can improve robustness in localization overcoming the accuracy degradation problem due to RF multipath and noise that are dominant in the conventional RSS measurement-based LTE localization scheme. The test results in urban areas demonstrate that the proposed surface correlation-based fingerprinting method has improved performance compared to the other conventional methods, thus proving to be a useful complementary method to the GNSS in urban areas.

## 1. Introduction

Recently, the rapid development of wireless communication technology has increased the dependency of human life on location-based services. Reliable localization technology is widely applied to various fields such as gaming, social networking service, industrial safety, as well as current vehicle navigation systems. The most commonly used technology for localization is the Global Satellite Navigation System (GNSS). It is obvious that GNSS is the most efficient and accurate in the outdoor environment. However, in complex urban areas or indoor environments, the accuracy of the GNSS is acutely degraded due to signal unavailability and the multipath fading of the satellite signals [1]. 

In order to mitigate the limitation of GNSS usage in urban areas, several wireless communication technologies for localization have been developed. In recent years, the Long Term Evolution (LTE) that has become widely used in the metropolitan areas is attracting attention as a means of localizing pedestrians or vehicles in these challenging areas [2,3,4]. The assisted-GNSS (A-GNSS) scheme uses assistance data provided by LTE to improve the initial operation of Global Positioning System (GPS)-based devices [5,6]. However, this scheme does not use LTE signals for localization. The enhanced Cell-ID (e-CID) technology was proposed to improve localization accuracy by adding Round Trip Time-based distance information to the existing Cell-ID method. However, its performance is limited depending on the size of the cell [7,8]. The Observed Time Difference of Arrival (OTDoA) technology defined in the 3GPP-LTE Release 9 standard is based on the Time Difference of Arrival (TDoA) method using the positioning reference signal of LTE. This technology estimates a location based on the hyperbolic trilateration of the TDoA measurement, Reference Signal Time Difference (RSTD) [9,10]. Since the OTDoA technology requires at least three Base Stations (BS) for trilateration, its usage is limited in areas with low BS density. Despite the above-mentioned LTE-based attempts to improve performance, such the technologies are still hard-pressed to satisfy the performance standards, such as accuracy, stability, and availability, required by various LBS services. 

In Non-Line-of-Sight (NLoS) environments, such as urban areas or indoors, a fingerprinting technology can be more effective than timing-based localization technology. The LTE Positioning Protocol requires that Received Signal Strength (RSS) information be provided as well as CID, Timing Advanced (TA), angle of arrival and channel status information [11,12]. Gundlegard et al. [13] proposed the use of Observed Time Differences (OTD) with RSS to improve the performance of a fingerprinting-based localization technology. Zhu et al. [14] developed a maximum likelihood-based fingerprinting method using RSS, RSTD, and TA of LTE system. It demonstrated that the proposed fingerprinting method is generally superior to the e-CID and OTDOA based localization method. Turkka et al. [15] developed a grid-based fingerprinting method using the RSS information of LTE and WLAN. The performance of LTE-based fingerprinting method was improved with the RSS information of WLAN. 

However, it has been reported that the fingerprinting-based localization technologies based on direct comparison of the measured RSS with fingerprinting database suffer from the lack of accuracy, stability, and robustness [16,17,18]. Received RF signals experience many distortion phenomena during propagation and vary consistently due to other RF signal` interference, multipath fading, and noise. The RF signal distortion and variation becomes more prominent in the areas where structures that prohibit direct propagation are densely located and diverse wireless applications are actively used. This is typical in the LTE signal generated in urban areas. In conventional fingerprinting technology that utilizes instantaneous RSS data, these distortion phenomena tend to increase ambiguity. Therefore, compensation methods for the fingerprinting-based localization technology to reduce such the RF signal`s distortion and instability problems must be devised.

To solve this problem, there have been researches integrating the Pedestrian Dead-Reckoning (PDR) with RF-based localization technology. The PDR can accurately estimate a location using a foot-mounted Inertial Measurement Unit (IMU) for a short period of time. By integrating a map matching algorithm, it is possible to operate accurately for a longer period of time [19,20]. However, in the case of a smartphone, it is difficult to expect a high-performance of the PDR due to the various activities of a pedestrian. Masiero et al. [21] developed a particle filter based integration method of inertial measurements, RSS of Wi-Fi, and map information using a smartphone. It is highly reliable and accurate to estimate the location of a pedestrian in an indoor environment. But it is still difficult to guarantee stable performance in the outdoor where the density of LTE BS and signal strength are very low due to the use of only instantaneous signal strength data. 

In this paper, we propose a new fingerprinting method based on the concept of Surface Correlation (SC) in the LTE environment of urban areas. The SC estimates a location by comparing the spatial RSS pattern data provided by the PDR algorithm with a fingerprinting database, rather than relying just on instantaneous RSS measurements as in the conventional fingerprinting method. This spatial RSS pattern not only improves the accuracy and stability of localization performance by reducing ambiguity but also increases the availability of the LTE-based localization technology. The contributions of this study are following:

- We develop an SC-based fingerprinting technology that computes a location through comparison of spatial RSS patterns, rather than instantaneous RSS data as applied in the conventional fingerprinting method, between the database and measurements.
- For comparison between the measurements and database, we develop methods for generating spatial RSS patterns from them according to the movement of a pedestrian.
- We obtain accurate and stable location information by using few LTE base stations even in error-prone urban areas.

The paper is organized as follows: Section 2 provides an overview of the state-of-the-art surface correlation approach to the proposed fingerprinting method. Section 3 describes the details of its application on the fingerprinting method. The performance of the proposed fingerprinting method is discussed in Section 4. Finally, Section 5 presents conclusions drawn in this work.

## 2. Algorithm Overview

**Figure 1:**
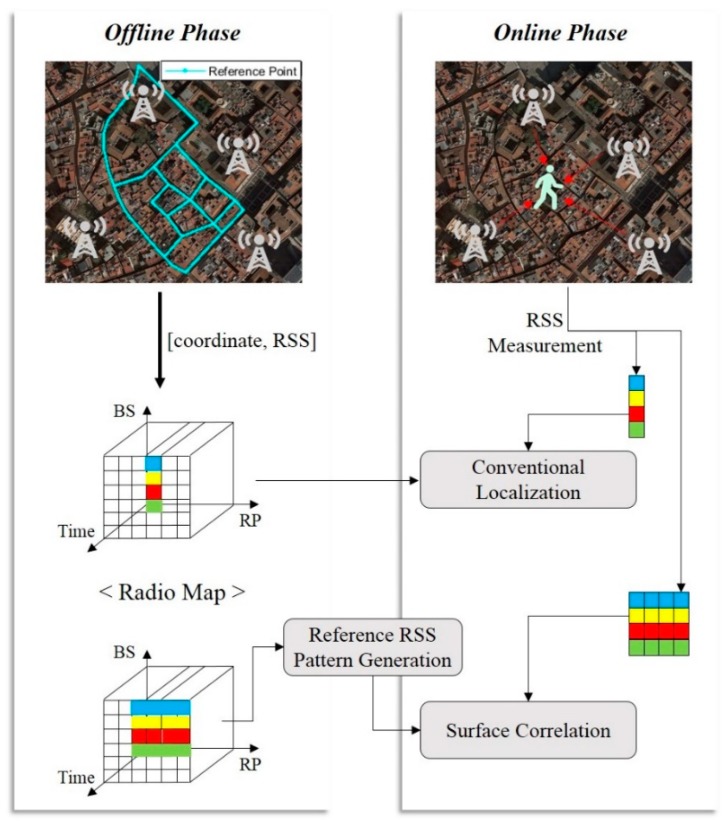
Overview of conventional and proposed fingerprinting methods.

Generally, a fingerprinting method involves an offline phase and an online phase. In the offline phase, a survey is performed to construct a fingerprinting database as displayed in Figure 1. This is achieved by storing the RSS of signals received from surrounding signal sources at a specified Reference Point (RP). In the online phase, a terminal device measures the RSS received from surrounding signal sources and estimates location by searching the RP with similar fingerprint. As shown in Figure 1, the RSS measurement in the conventional fingerprinting method is through instantaneous RSS data, which can increase ambiguity due to noise and distortion by the surrounding environment. While the effects of noise and distortion are low in areas with high RSS, it is impossible for stable and accurate localization due to the increased ambiguity in areas with very low RSS such as deep urban areas.

The SC method compares RSS patterns accumulated during movement with the fingerprinting database to estimate the location. The SC method can provide high performance (availability) regardless of the high and low areas of the LTE signal strength because it compares the spatial RSS variation pattern, not instantaneous data, with the reference pattern from the database. In this paper, we define the RSS pattern accumulated during movement, i.e. the spatial RSS pattern, as the *Surface*. We have already shown that the performance of the conventional fingerprinting method improves by comparing a one-dimensional fingerprinting database with spatial RSS patterns [22,23]. An urban area is a two-dimensional space with multiple roads combined, not just a simple one-dimensional space. Therefore, the SC method requires the generation of reference RSS patterns that match with a pedestrian`s route from the fingerprinting database for comparison between the measured spatial RSS pattern (*Surface*) and the database. To do this, it is required to construct a link-centered database rather than an RP-oriented database as seen in the conventional fingerprinting method. The SC method consists of four processes for localization: (1) domain conversion of RSS measurements; (2) generation of reference trajectory from a fingerprinting database; (3) correlation; (4) localization. 

## 3. Surface Correlation-based Fingerprinting Method

### 3.1. Fingerprinting Database

**Figure 2:**
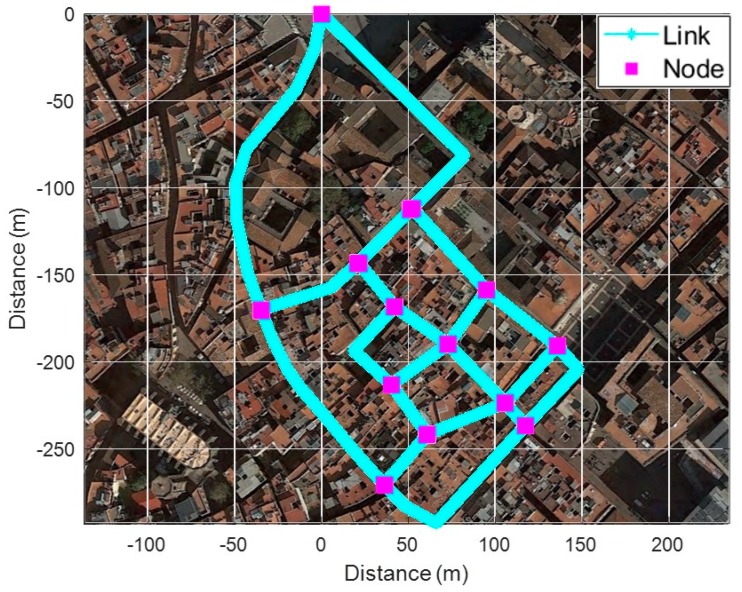
The link-node structure for fingerprinting database.

Generally, the fingerprinting database consists of the coordinates of RPs and RSS data from surrounding BS at the RP location. As mentioned above, the SC method compares spatial RSS patterns generated during pedestrian movement with the database; thus, it is necessary to generate a reference trajectory matching with the pedestrian’s route from the database. Therefore, it utilizes a database of ‘link-node’ structures to generate the reference trajectories dynamically. The link is generated based on crossroads and consists of a set of RPs as shown in Figure 2. The crosspoint connecting each link is defined as a node. The fingerprinting database can be expressed as follows:

(1) $$RP_{k}^{l}=\left[\left(x,y\right),\left\{\left(PCI_{1}:RSS_{1}\right),\cdots ,\left(PCI_{n}:RSS_{n}\right)\right\}\right]$$

where, $RP_{k}^{l}$ is the *k*th RP on the *l*th link, (*x*,*y*) is the coordinate of RP, $PCI_{n}$ is Physical Layer Cell ID (PCI) of *n*th BS, and *RSS_n_* is the received signal strength from *PCI_n_*, respectively. The reference trajectory, which matches the route of a pedestrian, is generated through a combination of $RP_{k}^{l}$.

### 3.2. Domain Conversion

**Figure 3:**
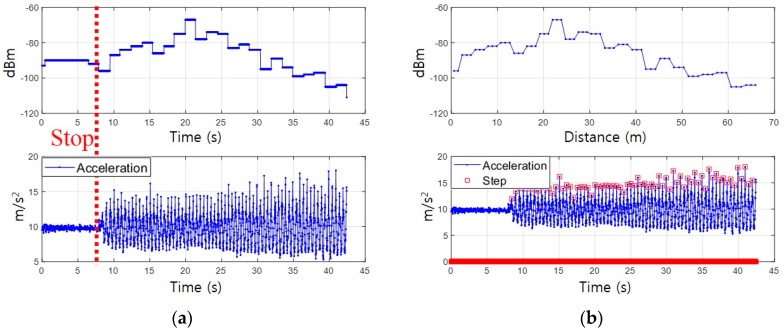
Domain conversion: (**a**) Received Signal Strength (RSS) pattern on time domain; (**b**) RSS pattern on the spatial domain.

RSS measurement is distributed in the time domain since it is measured at a certain time interval on a terminal device. Thus, it can be expressed as:

(2) $$\mathrm{Meas}=\left[\begin{array}{cc} \begin{array}{c} t_{1}\\ \vdots \\ t_{m} \end{array} & \begin{array}{ccc} RSS_{t_{1}}^{1} & \cdots & RSS_{t_{1}}^{n}\\ \vdots & \ddots & \vdots \\ RSS_{t_{m}}^{1} & \cdots & RSS_{t_{m}}^{n} \end{array} \end{array}\right]$$

where, $RSS_{t_{m}}^{n}$ is the received signal strength from *n*th BS at $t_{m}$. The conventional fingerprinting database consists of the RP coordinate and the RSS values at that point as Equation (1). The conventional fingerprinting methods compare the instantaneous RSS data ($RSS_{t_{m}}^{1:n}$) at time $t_{m}$ with the RSS vector on each RP, so it is possible to compute a location. Given that the RSS pattern measured on time domain cannot be compared with the RSS pattern on the database distributed in the spatial domain, the measured RSS data should be converted into a spatial domain. To do this, the proposed fingerprinting method utilizes the pedestrian step length estimation algorithm already proposed in an earlier study [24], where step length is modeled as a linear combination of the acceleration amplitude, walking frequency, and sway angle at every step. The SC method estimates the walking distance *d* by accumulating the step length and accumulates the measured RSS according to the walking distance as given in Figure 3. Thus, the *Surface* can be expressed as:

(3) $$S=\left[\begin{array}{c} S_{1}\\ \vdots \\ S_{m} \end{array}\right]=\left[\begin{array}{cc} \begin{array}{c} d_{1}\\ \vdots \\ d_{m} \end{array} & \begin{array}{ccc} RSS_{d_{1}}^{1} & \cdots & RSS_{d_{1}}^{1}\\ \vdots & \ddots & \vdots \\ RSS_{d_{m}}^{n} & \cdots & RSS_{d_{m}}^{n} \end{array} \end{array}\right]$$

where, *m* is the number of steps, *S* is the *Surface* at walking distance $d_{m}$ and $RSS_{d_{m}}^{n}$ is the received signal strength from *n*th BS at walking distance $d_{m}$. The proposed fingerprinting method estimates location by correlating the RSS pattern on the reference trajectory with the measured *Surface*. The reference RSS pattern generated from the fingerprinting database also forms a *Surface* as in Equation (3) by calculating the Euclidean distance between the adjacent RPs. 

### 3.3. Surface Correlation

**Figure 4:**
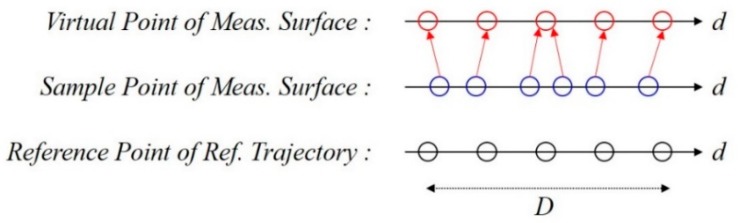
Generation of virtual *Surface* for the correlation process.

**Figure 5:**
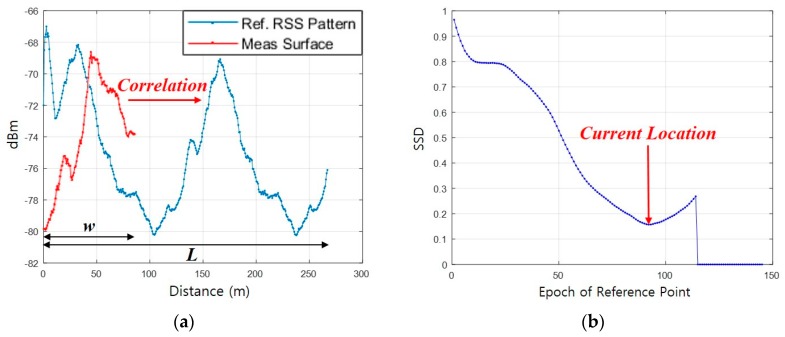
Correlation process: (**a**) Correlation between measured *Surface* and reference RSS pattern; (**b**) Surface similarity distance.

The Surface Correlation is the process of determining a location by calculating the similarity between the measured and reference RSS patterns. The similarity is expressed as a Surface Similarity Distance (SSD) and calculated as follows:

(4) $$\rho_{i}=\overset{L-w}{\underset{i=1}{\sum }}\overset{w}{\underset{j=1}{\sum }}\left\{\frac{\sqrt{\sum_{k=1}^{m}{\left(S_{j,k}^{Meas}-S_{i+j-1,k}^{DB}\right)}^{2}}}{m}\right\}$$

where *i* is the epoch of RP on a generated reference trajectory and *m* is the number of BS. $\rho_{i}$ is the SSD for *i*th calculation, $S^{Meas}$ is the measured *Surface*, and $S^{DB}$ is the RSS pattern on the reference trajectory. *L* is the number of RPs in the reference trajectory and *w* is the number of samples on the *Surface* during the time of walking distance *D*, that is, the number of estimated steps. To calculate the similarity distance, the number of RPs selected in the reference trajectory should be equal to the number of samples in the measured *Surface* during the period of walking distance *D,* which is not usually possible. This is because the step length of a pedestrian and the interval between the RPs is not equal. To solve this problem, we generate a virtual *Surface* equal to the number of RPs within distance *D* through linear spacing as shown in Figure 4. The virtual *Surface* is utilized for the calculation of SSD as shown in Figure 5a. The *i*th RP coordinate with the minimum $\rho_{i}$ is considered as the current location as shown in Figure 5b. 

### 3.4. Generation of Reference Trajectory

For accurate localization using the proposed surface correlation method, it is essential to generate a reference trajectory that matches the pedestrian’s route. This is because it is necessary to compare the measured RSS pattern with the reference RSS pattern that matches the movement route to make accurate localization possible. The reference trajectory is generated by combining links adjacent to the link where a pedestrian is currently located; therefore, it is very important to determine the link where the pedestrian is currently located. It is possible with substantial accuracy in an environment with good GNSS conditions, but quite difficult in a complex urban area. In this study, we determine the link where a pedestrian is currently located without GNSS. There are two steps to determine the required link: (1) Generation of reference trajectory in an initial phase; (2) Generation of reference trajectory after the initial phase.

#### 3.4.1. Generation of Reference Trajectory in Initial Phase

**Figure 6:**
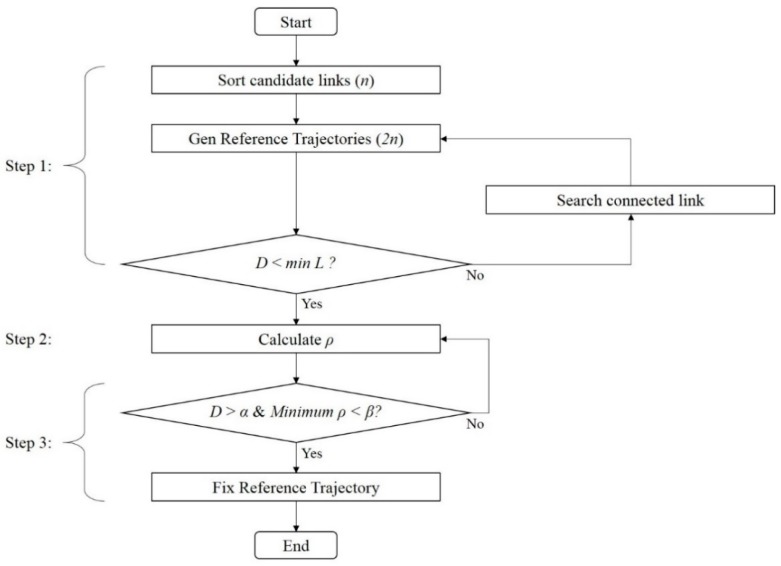
Flow chart for the generation of reference trajectory at the initial phase.

Since there is no location information in the initial phase of localization, the current link should be determined using only PCI information and RSS measurement from a terminal device. At first, the proposed fingerprinting method selects candidate links using only PCI information, and then generates reference trajectories using the selected links. A reference trajectory is fixed by correlation the measured RSS pattern and the RSS patterns on the generated reference trajectories. In other words, SSD is the criterion not only for determining the similarity between the RSS patterns, but also for selecting the actual trajectory where the current pedestrian is located among the generated multiple reference trajectories. The detail of this process is given in Figure 6.

**Step 1**: Generate reference trajectories as follows:

- Find and select *n* candidate links including measured PCI from the fingerprinting database.
- For each of the selected candidate links, generate bi-directional *2n* reference.
- If the estimated walking distance *D* is larger than the shortest reference trajectory *L* before checking *Minimum ρ < β*, generate new reference trajectories by combining links connected with the current reference trajectory. Only the newly generated reference trajectories matching with estimated turn event (left, right, or straight) from the Pedestrian Dead-Reckoning (PDR) are used for the correlation process.

**Step 2**: Calculate the SSD on each reference trajectory using Equation (4).

**Step 3**: When the pedestrian moves over a certain distance (α) and the SSD value is stable (*Minimum ρ < β*), the reference trajectory with the minimum SSD is fixed and the location is estimated on the fixed reference trajectory.

#### 3.4.2. Generation of Reference Trajectory after Initial Phase

**Figure 7:**
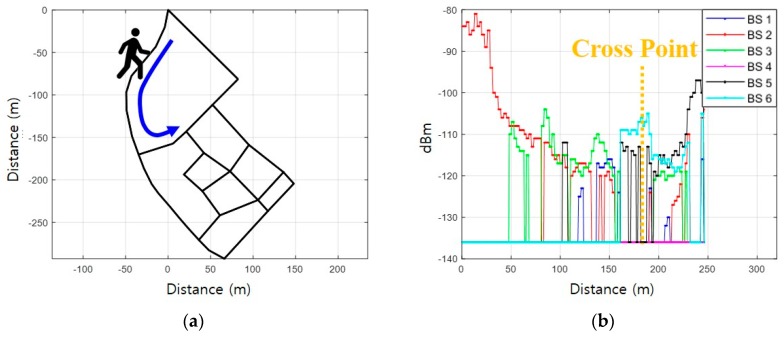
Movement scenario of a pedestrian: (**a**) Route; (**b**) Measured RSS during movement.

**Figure 8:**
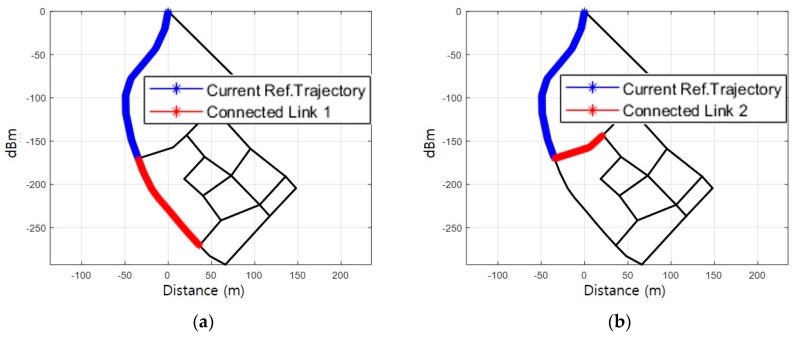

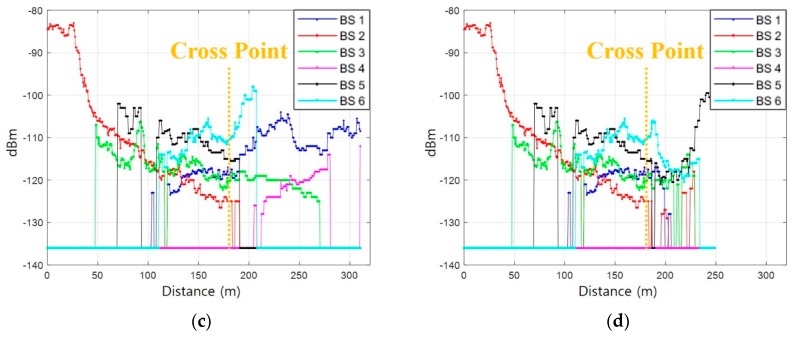
Generation of reference RSS pattern: (**a**) Regenerated reference trajectory 1; (**b**) Regenerated reference trajectory 2; (**c**) RSS pattern of reference trajectory 1; (**d**) RSS pattern of reference trajectory 2.

After the initial reference trajectory is fixed, a new reference trajectory is again generated by combining the current reference trajectory and the adjacent links. A reference trajectory is fixed for the moving direction and the route of the pedestrian by correlating the newly generated reference trajectories. Specifically, the walking direction is estimated by selecting a reference trajectory between the initial bidirectional links, not in the absolute direction from PDR. Let us assume that the pedestrian moves as shown in Figure 7a; measured *Surface* during the movement is shown in Figure 7b. The proposed fingerprinting method generates two reference trajectories by combining connected links with the current reference trajectory as shown in Figure 8a and b. Figure 8c and d show the respective reference RSS patterns on the generated reference trajectory.

**Figure 9:**
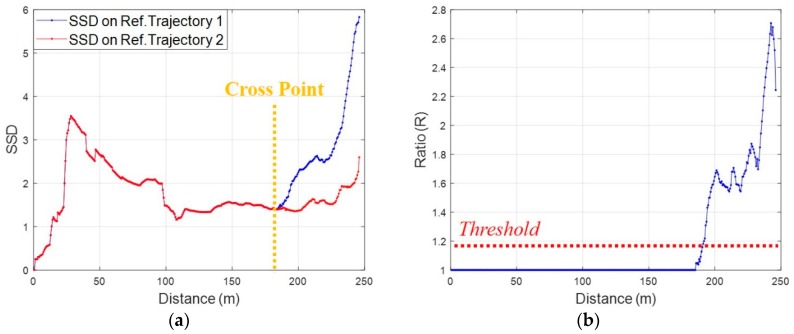
Fix a reference trajectory using a ratio between values of SSD: (**a**) SSDs of reference trajectories; (**b**) Ratio between SSDs.

In the initial phase, the proposed method fixes a reference trajectory based on the minimum SSD. After the initial phase, it fixes a reference trajectory based on the ratio of SSDs as shown in Equation (5) below:

(5) $$R=\frac{\rho_{1}}{\rho_{2}},\;\;\;\;if\;\rho_{1}>\rho_{2}$$

where $\rho_{2}$ is the minimum SSD between the reference trajectories and $\rho_{1}$ is the second smallest value of the SSD. The values of SSDs change after the pedestrian has passed a crossroad as shown in Figure 9a. The reason why the ratio *R* has a value 1 before the crossroad is that the reference trajectory 1 and the reference trajectory 2 have a common current reference trajectory. As shown in Figure 9b, after the crossroad, *R* increases because of the difference between the measured RSS pattern and the reference RSS patterns. Therefore, it is possible to fix a reference trajectory that matches the pedestrian’s walking route using only a threshold for the ratio of SSDs. After fixing the reference trajectory, new reference trajectories are generated by combining links with the fixed reference trajectory again.

### 3.5. Localization

**Figure 10:**
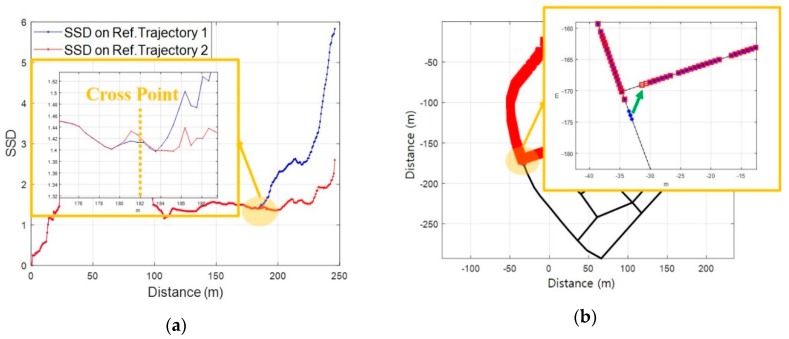
The problem of localization using the minimum SSD at a cross point: (**a**) SSD of each reference trajectory at a crossroad; (**b**) Localization error and compensation using Pedestrian Dead-Reckoning (PDR) turn event.

As mentioned before, the SC method selects the RP with the minimum SSD on the fixed reference trajectory as the current location. Figure 10b shows the result of localization according to the calculated similarity distance in Figure 9 and Figure 10a. As shown in Figure 10b, it can be seen that a localization error occurs at the crossroad when the RP with the minimum similarity distance value is selected. A crossroad is a relatively open space; therefore, the RSS patterns between the reference trajectories are very similar. This increases ambiguity when compared with the measured *Surface*, resulting in a localization error. The proposed method gives a priority to the reference trajectory that matches a turn event from the PDR to reduce ambiguity at a crossroad, which means that the location is estimated from the reference trajectory that corresponds to a turn event from the PDR. However, various motions of the pedestrian can cause a false turn event reading of the PDR. To prevent this, the proposed method continuously calculates SSDs for all the generated reference trajectories until one of them is fixed. If the fixed reference trajectory is different from the turn event matched trajectory, localization is processed on the fixed reference trajectory. In this paper, we set a threshold to fix a reference trajectory as 1.16 to reduce the error of the PDR turn event at a crossroad.

**Figure 11:**
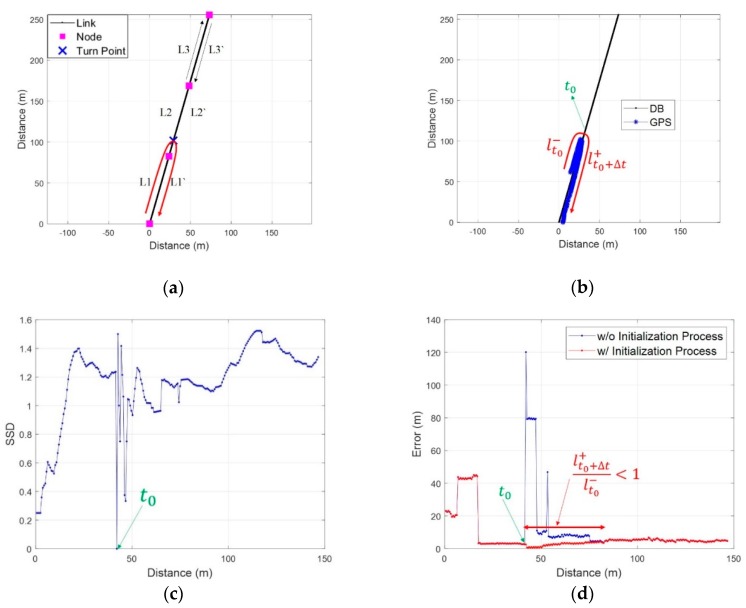
The localization of the Surface Correlation (SC) method in case of a turn back event: (**a**) Links for the generation of a reference trajectory; (**b**) Test route for a turn back event; (**c**) SSD; (**d**) Localization error by comparison with GPS.

In addition to the left and right turn of a pedestrian, the event of turning back on a link should also be considered. The SC method generates a reference trajectory through a combination of links. Therefore, when the pedestrian is moving as shown in Figure 11a, the SC method computes the location using a reference trajectory comprising a combination of *L1*, *L2*, and candidate *L3* before the turn back event. It is possible to generate a reference trajectory by combining *L1*, *L2*, *L2’* and *L1’*. But, in this case, it is impossible to perform an accurate comparison between *Surfaces* because the reference trajectory contains RPs that the pedestrian did not actually pass. It is very difficult to determine an exact RP at the time of turn event; thus, it is also impossible to divide *L2* and *L2’* to generate a reference trajectory.

For these reasons, the SC method performs an initialization process that resets both the measured *Surface* and the current reference trajectory when a turn back event occurs as shown in Figure 11b. However, the resetting of the measured *Surface* may cause ambiguity as in conventional fingerprinting methods as Figure 11c and d. The length of the *Surface* is a very important factor in identifying the reliability of the location information through SC, along with the SSD. To mitigate the unstable performance due to the ambiguity after the turn event, the SC method fixes the reverse component of a current link as the reference trajectory and an RP as a start point when the turning event occurs. Then, it determines the location using length of the *Surface* until its length ($l_{t_{0}+\Delta t}^{+}$) is greater than the length of the previous *Surface* ($l_{t_{0}}^{-}$). Let $t_{epoch}$ denote the fixed RP where the turn event has occurred and denote the total number of RPs on the reference trajectory. If $l_{t_{0}+\Delta t}^{+}/l_{t_{0}}^{-}\geq 1$, the SC method determines the location as an RP_a*_ with the minimum SSD as presented below:

(6) $$a^{*}=\underset{1\leq a\leq t_{epoch}\leq m_{r}}{\arg\;\min}\rho_{\left[1,t_{epoch}\right]}$$

## 4. Experiments and Results

### 4.1. Test Environment

**Figure 12:**
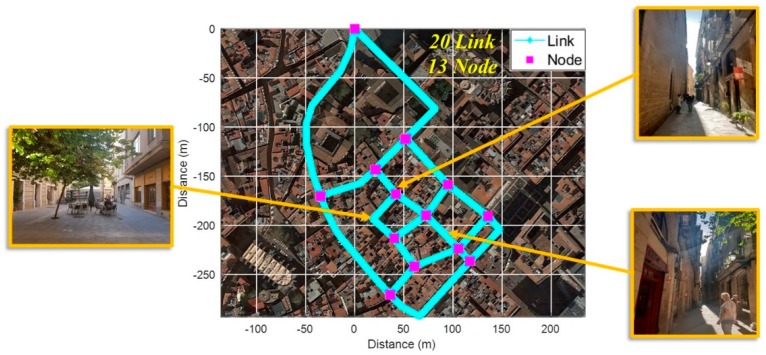
Test Site.

To verify the proposed fingerprinting method, we performed tests in Barcelona’s old districts with a common commercial smartphone as shown in Figure 12. Before the test, we constructed a fingerprinting database consisting of 20 links and 13 nodes through several surveys of the test area. 

**Figure 13:**
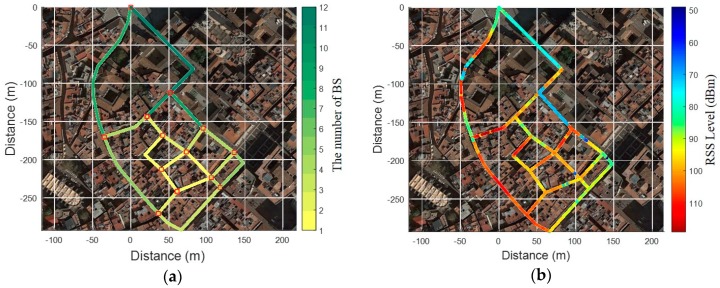
The condition of the Long-Term Evolution (LTE) environment in the test site: (**a**) The number of available BSs at each link; (**b**) The maximum RSS at each RP.

**Figure 14:**
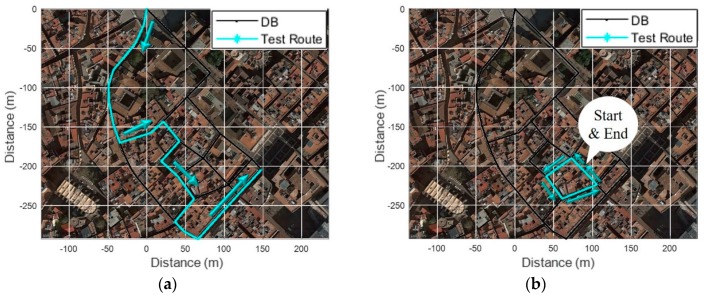
Test scenarios: (**a**) Open space into narrow alley; (**b**) Narrow alley to alley.

As shown in Figure 13a, four BSs are available on average on each link. Over 10 BSs were available in a square or a relatively wide alley, but only one BS was available in narrow alleys. In addition, measured RSS detected as very low in most RPs, as shown in Figure 13b. As shown in Figure 14a and b, tests were performed in two scenarios as follows: (1) From an open space into a narrow alley; (2) From the narrow alley to an alley. In order to analyze the performance in each scenario, localization results from PDR were corrected by applying a map-matching algorithm and it was considered as a true location. Collected data from the smartphone were as follows:

- Inertial Measurement Unit (IMU): 40 Hz
- PCI & RSS of LTE: 0.5 Hz
- GNSS: 1 Hz

### 4.2. Performance Analysis according to the Length of Surface

The length of the *Surface* is the most important factor determining the performance of the proposed fingerprinting method. As its length increases, the performance of the proposed method becomes more robust against signal noise and distortion, and the ambiguity can be reduced while performing a comparison with the RSS pattern. On the other hand, the following problems may occur as the length of the *Surface* increases:

- Drift error of walking distance from PDR
  The walking distance is calculated by accumulating the step lengths from the PDR, and the *Surface* is constructed based on this distance. Drift error of the walking distance induces a localization error when comparing the measured *Surface* with the reference RSS pattern.
- Sensitivity degradation in fixing reference trajectory at crossroad
  As the length of *Surface* increases, the proportion of the RSS pattern to the common reference trajectory is higher than that of the RSS pattern after a crossroad. As a result, the similarity of RSS patterns between reference trajectories increases and the ratio of correlation coefficient becomes smaller. This serves as a delay in fixing the reference trajectory, which may lead to a problem of fixing the previous reference trajectory beyond the next crossroad.

**Figure 15:**
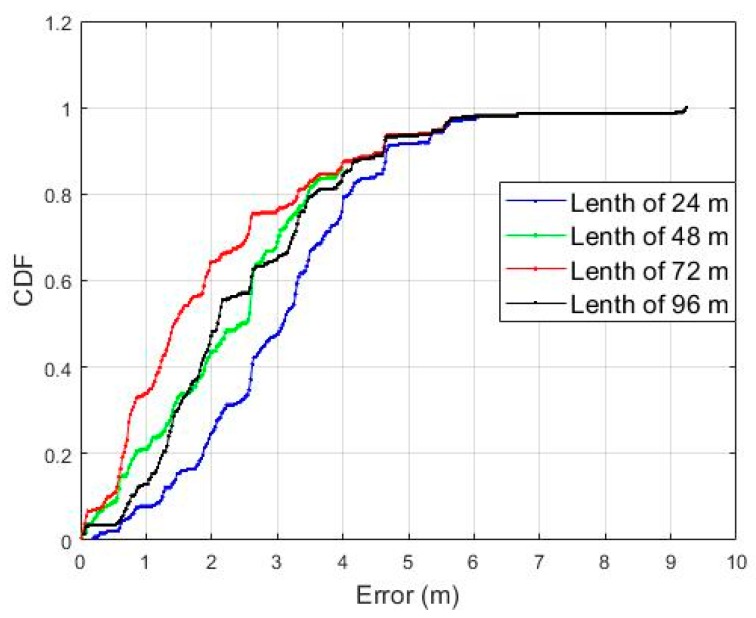
The CDF of localization error according to the length of the *Surface.*

We selected the optimal length by comparing the localization errors for different lengths of *Surface*. In order to correctly analyze the accuracy according to the length of the *Surface*, localization was performed only for the reference path in Figure 8b while the pedestrian moved as shown in Figure 7a. As shown in Figure 15, accuracy improves as the length of the *Surface* increases up to 72 m.

The accuracy decreases as the length increases more than 72 m. Therefore, we selected 72 m as the maximum length of the Surface in this study. In addition, a reference trajectory was set to be combined with up to three links considering the length of the Surface and that of the shortest link. This limitation also has the effect of bounding the increase of computation complexity in the comparison between measured Surface and reference patterns.

### 4.3. Test Result from Open Space into Narrow Alley

**Figure 16:**
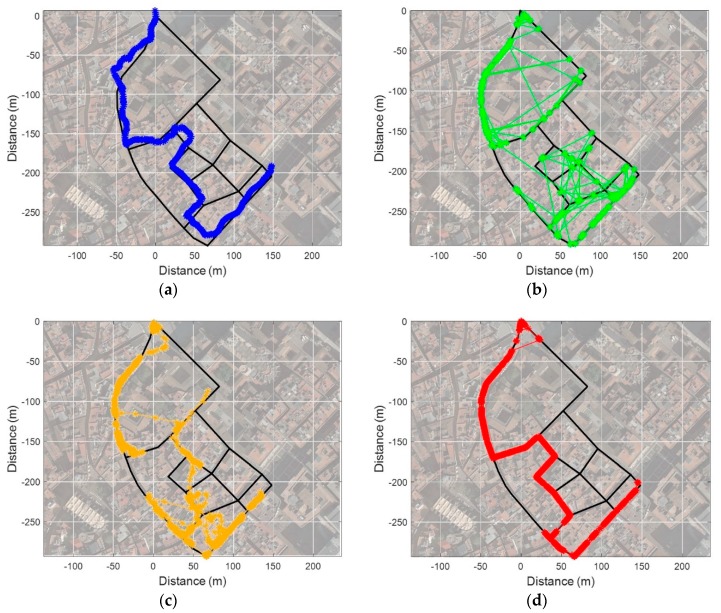
Scenario 1 test results: (**a**) GPS; (**b**) Conventional fingerprinting (kNN); (**c**) Particle Filter with PDR; (**d**) SC.

**Figure 17:**
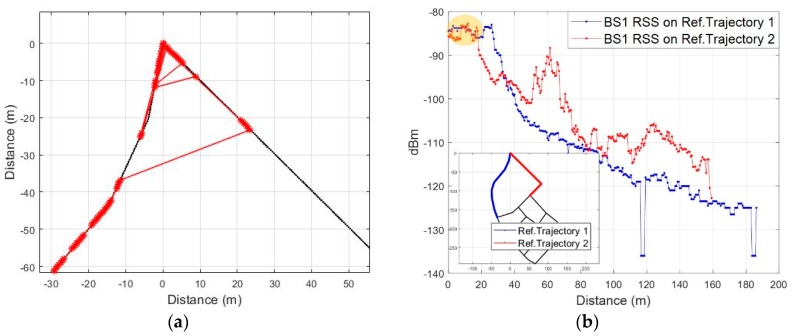
Initial localization error: (**a**) Result of initial localization; (**b**) RSS pattern of reference trajectories from six Base Stations (BSs).

**Figure 18:**
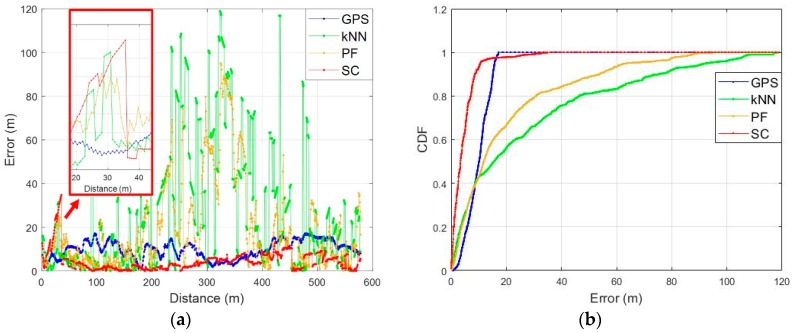
Localization error on test scenario 1: (**a**) Localization error according to the walking distance; (**b**) CDF of the localization error.

In this scenario, a test was performed in an open space with good GNSS conditions and LTE environment as shown in Figure 13. To verify the performance of the proposed fingerprinting method, we compared its result with the GPS, the conventional fingerprinting method (kNN), and a Particle Filter (PF) as shown in Figure 16. We set the k as 5 to estimate the location from the kNN fingerprinting method. We utilized the heading and stride information from PDR on a resampling process of the PF for the particle propagation. Additionally, 150 particles were used to compute the location. As shown in Figure 16a–d, the kNN-based fingerprinting and PF show a relatively stable performance in a region with good LTE environment. However, in a region with poor LTE signal environment, the performance is very unstable due to increased ambiguity. The GPS showed a relatively stable performance when entering a narrow alley after convergence of performance in open space. However, it can be seen that a multipath effect on the satellite signal in the alley deviates it substantially from the actual route. The SC method shows a very stable performance on the overall test route. However, at the initial stage of the test, we confirmed that the location was matched to a different link than the actual link, just like the kNN as in Figure 17a. As shown in Figure 17b, this is because the RSS pattern for the two reference trajectories is very similar near the starting point and sufficient RSS pattern is not accumulated for ambiguity reduction. It can be seen that the localization result is very stable after 35 m point where the RSS pattern is accumulated sufficiently and ambiguity decreases. Except for the initial phase, we can confirm that the SC-based fingerprinting method is very stable and accurate in most areas as demonstrated in Figure 18a,b. The Root Mean Square Error (RMSE) of the GPS is 10.86 m and of the conventional fingerprinting method is 41.22 m, respectively. The RMSE of the PF is 28.59 m while the surface correlation method has an RMSE of 4.67 m except for the initial error.

### 4.4. Test Result from Narrow Alley to Alley

**Figure 19:**
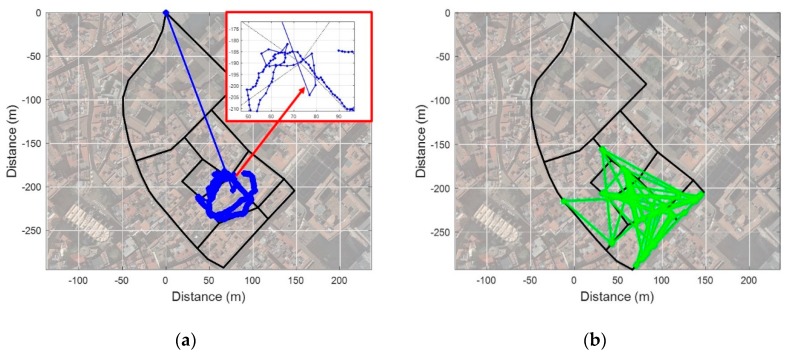

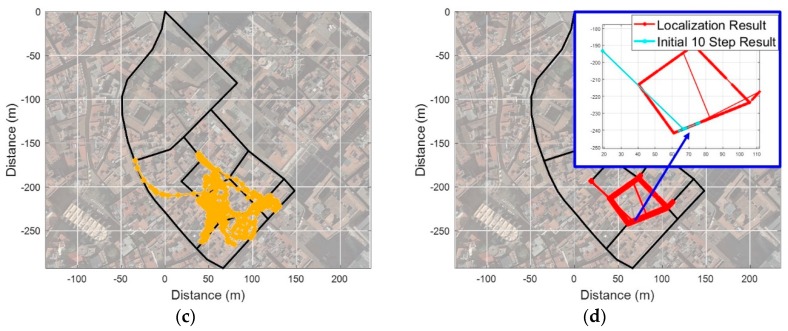
Scenario 2 test results: (**a**) GPS; (**b**) Conventional fingerprinting (kNN); (**c**) Particle Filter with PDR; (**d**) SC.

**Figure 20:**
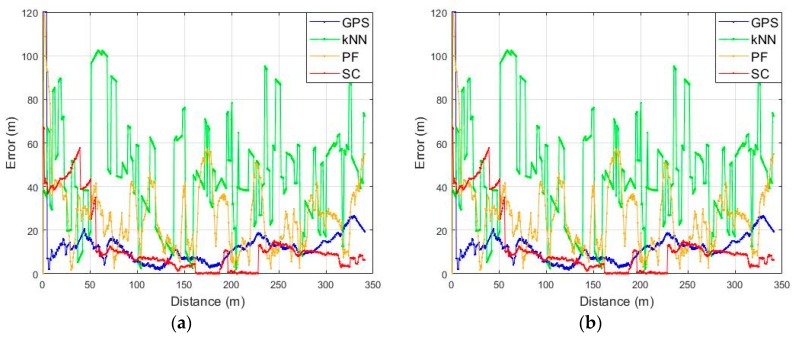
Localization error on test scenario 2: (**a**) Localization error according to the walking distance; (**b**) CDF of the localization error.

In order to verify the performance in an area where the LTE environment is poor, the test was performed with the same route as shown earlier in Figure 14b. This test area had only one available BS as seen in Figure 13a, and also had poor GPS signal conditions as shown in Figure 19a. The estimated initial location from GPS was at origin because it did not receive satellite signal at the beginning of the test. In the case of the kNN and the PF, localization was almost impossible in areas where the LTE environment was very poor with a few BSs while the SC shows a stable and accurate performance as seen in Figure 19b–d. In test scenario 2, there was only one available BS; therefore, 24 more steps were needed to stabilize the performance than in test scenario 1 as shown in Figure 20. The RMSE of the GPS is 18.52 m and the kNN is 53.04 m, respectively. The PF shows RMSE at 31.73 m while the SC-based fingerprinting method shows it at 7.85 m except for the initial phase error. The test results for the above two scenarios are summarized in Table 1, and they can confirm that our proposed method shows better or similar performance with GPS even in a poor LTE environment. Especially, the availability of the RF coverage is definitely improved by utilizing the accumulated RSS pattern visible in the results of scenario 1 and scenario 2 with different LTE environmental conditions. This implies that it is possible to maintain a high performance with only a small number of BSs using the proposed fingerprinting method even in urban areas.

## 5. Conclusions

Accurate localization in urban areas still is a challenging task even with GNSS. In this paper, we proposed a surface correlation based fingerprinting method using LTE signals in urban areas. The surface correlation method compares the spatial RSS pattern of the pedestrian movement with a fingerprinting database. For this comparison of patterns, it generates a reference trajectory from the fingerprinting database that matches the walking path of a pedestrian. Accuracy and stability of the localization performance can be improved through the comparison between the RSS patterns. Moreover, the proposed method provides spatial discrimination for a signal of very low strength, so the scope of applying localization is very wide. The test results show that the performance, measured in terms of accuracy, stability, and robustness, of the proposed method is similar or better than that of GPS even in the narrow alleys of an urban area. Additionally, it is expected that its combination with GNSS will make it more seamless and make accurate localization possible in urban areas. The surface correlation method is applicable not only to LTE, but also to most RF signals that can be measured in signal strength such as Wi-Fi, Bluetooth, and 5G; hence, it can be applied not only to urban areas but also to various environments, such as underground or indoors. 

## Figures and Tables

**Table 1:** Comparison of the performance.

Scenario	GPS	kNN	PF	SC
1	10.15 m (50%) 13.99 m (80%)	15.47 m (50%) 46.53 m (80%)	11.93 m (50%) 30.7 m (80%)	3.42 m (50%) 7.19 m (80%)
2	11.69 m (50%) 15.73 m (80%)	44.99 m (50%) 62.63 m (80%)	25.98 m (50%) 38.26 m (80%)	8.59 m (50%) 13.28 m (80%)

## References

[1] Ben-Moshe B., Elkin E., Levi H., Weissman A. Improving Accuracy of GNSS Devices in Urban Canyons. Proceedings of the 23rd Canadian Conference on Computational Geometry.

[2] Del Peral-Rosado J.A., López-Salcedo J.A., Seco-Granados G., Zanier F., Crisci M. Analysis of Positioning Capabilities of 3GPP LTE. Proceedings of the 25th International Technical Meeting of the Satellite Division of the Institute of Navigation 2012 (ION GNSS 2012).

[3] Seco-Granados G., Navarro-Gallardo M., Mıguez-Sanchez J., Lopez-Salcedo J.A., Del Peral-Rosado J.A., Garcıa-Molina J.A., Zanier F., Crisci M., I Castillo R.E. Performance Analysis of Hybrid GNSS and LTE Localization in Urban Scenarios. Proceedings of the 8th ESA Workshop on Satellite Navigation Technologies and European Workshop on GNSS Signals and Signal Processing.

[4] Mahyuddin M.F.M., Isa A.A.M., Zin M.S.I.M., H A.M.A., Manap Z., Ismail M.K. (2017). Overview of Positioning Techniques for LTE Technology. JTEC.

[5] Mike T., Ewald Z. (2013). LTE Location Based Technology Introduction.

[6] Kangas A., Wigren T. Location Coverage and Sensitivity with A-GPS. Proceedings of the URSI International Symposium on Electromagnetic Theory.

[7] Kunczier H., Anegg H. Enhanced Cell ID based Terminal Location for Urban Area Location based Applications. Proceedings of the First IEEE Consumer Communications and Networking Conference.

[8] Wigren T. (2007). Adaptive Enhanced Cell-ID Fingerprinting Localization by Clustering of Precise Position Measurements. IEEE Trans. Veh. Technol..

[9] Liu J., Feng S. RSTD Performance for Small Bandwidth of OTDOA Positioning in 3GPP LTE. Proceedings of the 2013 IEEE 78th Vehicular Technology Conference (VTC Fall).

[10] Zhang T., Xiao D., Cui J., Luo X. A Novel OTDOA Positioning Scheme in Heterogeneous LTE-Advanced Systems. Proceedings of the 2012 3rd IEEE International Conference on Network Infrastructure and Digital Content.

[11] Evolved Universal Terrestrial Radio Access (E-UTRA) LTE Positioning Protocol (LPP). 3GPP TS 36.355. https://www.3gpp.org/ftp/Specs/archive/36_series/36.355/.

[12] Evolved Universal Terrestrial Radio Access (E-UTRA) LTE Positioning Protocol A (LPPa). 3GPP TS 36.455. https://www.3gpp.org/ftp/Specs/archive/36_series/36.455/.

[13] Gundlegard D., Akram A., Fowler S., Ahmad H. Cellular Positioning using Fingerprinting based on Observed Time Differences. Proceedings of the 2013 International Conference on Smart Communications in Network Technologies (SaCoNeT).

[14] Zhu J., Luo X., Chen D. Maximum Likelihood Scheme for Fingerprinting Positioning in LTE System. Proceedings of the 2012 IEEE 14th International Conference on Communication Technology.

[15] Turkka J., Hiltunen T., Mondal R.U., Ristaniemi T. Performance Evaluation of LTE Radio Fingerprinting using Field Measurements. Proceedings of the 2015 International Symposium on Wireless Communication Systems (ISWCS).

[16] Liu H., Darabi H., Banerjee P., Liu J. (2007). Survey of Wireless Indoor Positioning Techniques and Systems. IEEE Trans. Syst. Man Cybern. Part C.

[17] Torres-Sospedra J., Moreira A. (2017). Analysis of sources of large positioning errors in deterministic fingerprinting. Sensors.

[18] Deng Z., Yu Y., Yuan X., Wan N., Yang L. (2013). Situation and Development Tendency of Indoor Positioning. China Commun..

[19] Ascher C., Kessler C., Wankerl M., Trommer G.F. Dual IMU Indoor Navigation with Particle Filter based Map-Matching on a Smartphone. Proceedings of the 2010 International Conference on Indoor Positioning and Indoor Navigation (IPIN).

[20] Klepal M., Beauregard S. A Backtracking Particle Filter for Fusing Building Plans with PDR Displacement Estimates. Proceedings of the 2008 5th Workshop on Positioning, Navigation and Communication (WPNC).

[21] Masiero A., Guarnieri F., Pirotti F., Vettore A. (2014). A Particle Filter for Smartphone-Based Indoor Pedestrian Navigation. Micromachines.

[22] Shin B., Yu B., Bang J., Kee C., Lee T. WiFi based Robust Positioning System in Large Scale and Weak Signal Environment. Proceedings of the 30th International Technical Meeting of the Satellite Division of the Institute of Navigation.

[23] Shin B., Jeon S., Lee J.H., Kee C., Lee T. Precise Localization Technology of Mobile Phone on a Vehicle in Tunnel using LTE Signal based Surface Correlation. Proceedings of the 31st International Technical Meeting of the Satellite Division of the Institute of Navigation (ION GNSS+ 2018).

[24] Lee J.H., Shin B., Lee S., Park J., Kim J., Lee T. A Step Length Estimation based on Motion Recognition and Adaptive Gait Cognition using as Smartphone. Proceedings of the 27th International Technical Meeting of the Satellite Division of the Institute of Navigation.

